# Perfect Absorption Efficiency Circular Nanodisk Array Integrated with a Reactive Impedance Surface with High Field Enhancement

**DOI:** 10.3390/nano10020258

**Published:** 2020-02-02

**Authors:** Mohamad Khoirul Anam, Sangjo Choi

**Affiliations:** Department of Electrical Engineering, University of Ulsan, Ulsan 44610, Korea; anamulsan201@mail.ulsan.ac.kr

**Keywords:** infrared (IR) absorber, reactive impedance surface, electric field enhancement factor, absorption efficiency

## Abstract

Infrared (IR) absorbers based on a metal–insulator–metal (MIM) have been widely investigated due to their high absorption performance and simple structure. However, MIM absorbers based on ultrathin spacers suffer from low field enhancement. In this study, we propose a new MIM absorber structure to overcome this drawback. The proposed absorber utilizes a reactive impedance surface (RIS) to boost field enhancement without an ultrathin spacer and maintains near-perfect absorption by impedance matching with the vacuum. The RIS is a metallic patch array on a grounded dielectric substrate that can change its surface impedance, unlike conventional metallic reflectors. The final circular nanodisk array mounted on the optimum RIS offers an electric field enhancement factor of 180 with nearly perfect absorption of 98% at 230 THz. The proposed absorber exhibits robust performance even with a change in polarization of the incident wave. The RIS-integrated MIM absorber can be used to enhance the sensitivity of a local surface plasmon resonance (LSPR) sensor and surface-enhanced IR spectroscopy.

## 1. Introduction

The potential of infrared (IR) absorbers to achieve high efficiencies by controlling their shape and geometry has been the subject of intensive research [1]. Specifically, metal–insulator–metal (MIM) absorbers offer simple structures and the ability to realize high absorption using out-of-plane near-field coupling [2,3,4,5]. In a MIM absorber, a periodic array of metallic structures comprising square or circular patches is patterned on a dielectric spacer grown on a bottom metallic reflector. For perfect absorption, the dielectric spacer should be (~λ/100) thinner than the resonant wavelength because a thin spacer gives rise to strong magnetic dipole resonance and allows for an effective loop current from the opposing currents on the patch and the reflector [6,7,8]. Oscillating charges due to localized surface plasmon polaritons (LSPPs) on the sides of the patch and the imaged charges on the reflector excite a pair of antiparallel electric dipoles [9,10]. The magnetic and electric energy stored by the dipole resonance modes and metallic loss effectively capture the incident power of the incoming wave, achieving perfect absorption [6,7,8]. Because the absorption behavior caused by confined LSPPs from the MIM absorber is sensitive to a surrounding medium change even at the nanometer scale, MIM absorbers have been considered for sensor applications, such as thermal IR sensors [11,12], gas sensors [13], biosensors [14], localized surface plasmon resonance (LSPR) sensors [15], and surface-enhanced IR spectroscopy [16,17,18].

MIM absorbers with various metallic patch shapes using the ultrathin spacer for perfect absorption have been reported [6,7,8,19,20,21]. A gold nanostripe-based MIM with a dielectric spacer thickness of ~λ/530 achieved a 90% absorption rate near a wavelength of 1.5 μm [20]. In a similar spectrum, near-perfect absorption of 95% was achieved using a 10 nm thick grounded Al_2_O_3_ substrate with circular, square, and triangular patches [21]. However, MIM absorbers with ultrathin spacers suffer from low field enhancement (<100) [20,21], which is defined as the ratio between the incident field intensity and the excited field near the structure (E/E_0_), because opposing currents on the patch and the reflector that are too close cancel each other.

One way to increase the field enhancement of a MIM absorber is to control the ratio between the metal patch area and the unit cell area (the filling factor) [21,22,23]. For that purpose, the filling factor is set to be lower than 10% to maintain a collective effect in a patch array [22,23]. In [21], a circular disk array on a 10 nm thick grounded Al_2_O_3_ spacer with a high filling factor of 19.6% resulted in a field enhancement of 76. A similar nanodisk array on a grounded SiO_2_ spacer (23 nm thick) with a lower filling factor of 2.18% achieved a higher field enhancement factor of 85 at a wavelength of 860 nm [22]. This implies that changing the filling factor from the MIM absorber for a given ultrathin spacer cannot boost field enhancement significantly beyond 100 due to the aforementioned cancelation effect.

For array cases using relatively thicker grounded spacers or those without a ground, controlling the filling factor can also increase the absorption rate or extinction ratio [24,25,26,27,28,29,30,31,32,33,34]. Previous studies suggest that the maximum interaction occurs when in-phase coupling is maintained between scattered waves due to the array and LSPPs of each element [27,28,29]. Because the peak spectrum of LSPPs of each element in a MIM absorber depends on spacer thickness, both the thickness and the filling factor should be considered to maximize absorption and field enhancement at a given wavelength.

In this paper, we present a new IR absorber structure that can overcome the limitation of low field enhancement in ultrathin spacer-based MIM absorbers. The proposed structure maintains perfect absorption from impedance matching with the surrounding medium by utilizing a reactive impedance surface (RIS). The RIS is a metallic patch array on a grounded dielectric substrate that can alter its surface impedance, unlike a common metallic reflector. Previously, the RIS has been used for an antenna ground plane to improve antenna performance at microwave frequencies by offsetting the near-field capacitive feature through engineered surface impedance [35,36]. In this study, the metal structure on the top of the absorber uses a circular disk array, and the RIS compensates for the capacitive nature of the disk. To the best of our knowledge, this is the first attempt to use a RIS to improve the performance of a MIM-based IR absorber. Numerical simulations achieved an absorption efficiency of 98% and a high electric field enhancement factor of 180 at 230 THz (at a wavelength of 1.3 μm) using a circular nanodisk with a filling factor of 4.33% and without an ultrathin dielectric spacer. The value of the electric field enhancement from this structure was much higher than the maximum enhancement value of near 85 associated with circular nanodisks with grounded ultrathin spacers [21,22,23,31,32,33]. We further demonstrate a polarization-independent property of the proposed absorber by illuminating two different linearly polarized incident waves. The superior performance of the proposed IR absorber can be used to enhance the sensitivity of sensors, including LSPR sensors and surface-enhanced IR spectroscopy.

## 2. Simulation Methods

Our numerical simulations employed high-frequency structure simulator (HFSS) based on the finite element method. In the MIM absorber design, the metallic elements and insulators were gold and silicon dioxide (SiO_2_), respectively. For proper modeling in the near-IR region, frequency-dependent dielectric constant values for SiO_2_ from previous experimental studies were used [37,38]. The permittivity ε(ω) and conductivity σ(ω) of gold in the IR region were taken from the Drude model using the equations $\varepsilon \left(\omega \right)=1-\left[\left(\omega_{p}{}^{2}\tau^{2}\right)/\left(1+\omega^{2}\tau^{2}\right)\right]$ and $\sigma \left(\omega \right)=\left[\left(\varepsilon_{0}\omega_{p}{}^{2}\tau \right)/\left(1+\omega^{2}\tau^{2}\right)\right]$, with plasma frequency (ω_p_) and scattering time (τ) set to 2π × 2080 × 10^12^ rad/s and 18 fs [39,40], respectively.

**Figure 1:**
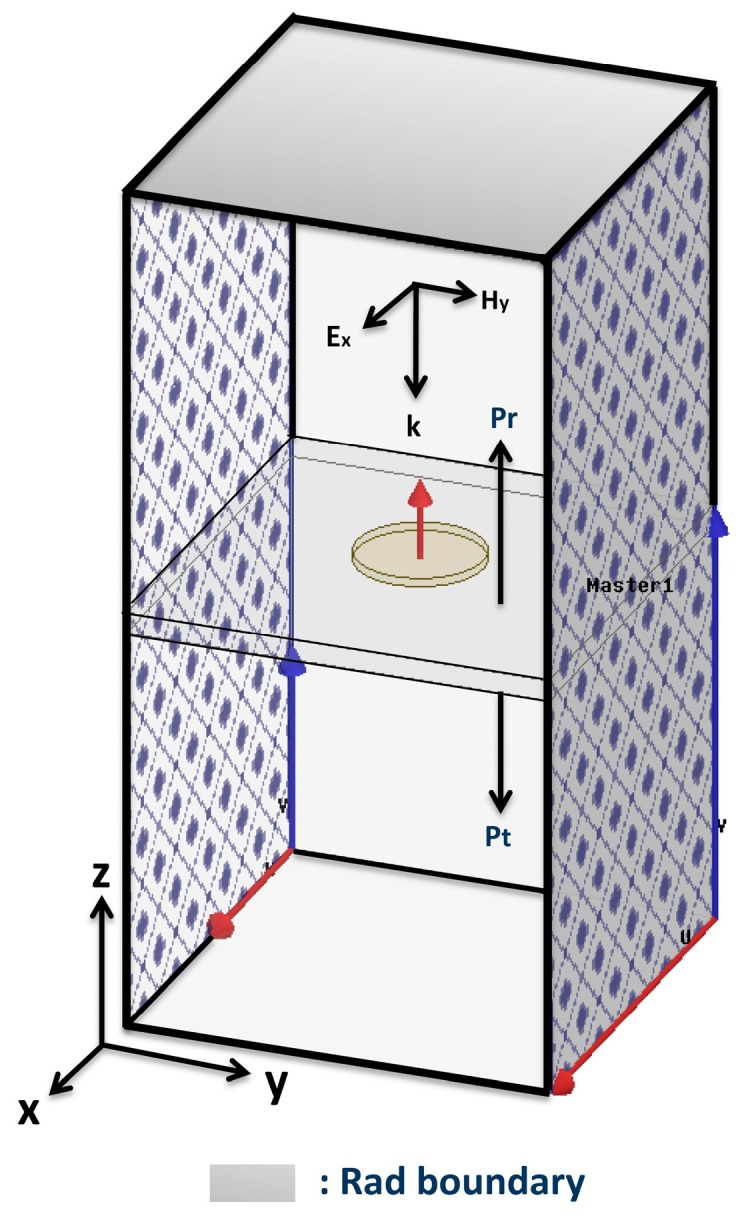
Schematic view of the circular nanodisk array simulation using a periodic boundary condition. Master and slave boundaries are used along the x–z and y–z planes, and the radiation boundaries are assigned on the top and bottom of the simulation boundary box.

All outer boundaries of the air box for the single nanodisk simulations were set by the radiation boundary. Meanwhile, an infinite array of circular nanodisks were realized by the periodic boundary condition (PBC) along the y–z and x–z planes and radiation boundaries at the top and bottom, as illustrated in Figure 1. The structure was then illuminated with an x-polarized incident plane wave in a normal direction (wave vector *k* is along the negative z-axis). The absorption rate of the circular nanodisk array was calculated using the equation A = 1 − (P_r_/P_i_) – (P_t_/P_i_), where P_r_, P_t_, and P_i_ are the reflected, transmitted, and incident power, respectively. The electric field enhancement factor was calculated at a hot spot 1 nm below the nanodisk edge [21,41]. Field enhancement was defined as the ratio between the magnitudes of the electric fields at the measurement point and the incident electric field (E/E_0_) [21,23,31,42,43].

## 3. Result and Discussions

First, we designed a single circular nanodisk on a grounded SiO_2_ substrate and selected a non-ultra-thin thickness for the SiO_2_ and a disk diameter that produced maximum field enhancement at 230 THz. Second, a two-dimensional array using the optimum single circular nanodisk was designed, and the filling factor was optimized to maximize field enhancement. In simulations, the filling factor was tuned by manipulating the distance between the antenna elements (array pitch). Here, we note that the designed nanodisk array could not achieve near-perfect absorption because the SiO_2_ was not ultrathin. Third, we designed a metallic patch array as a RIS that can fit under the unit cell area of a single circular nanodisk set by the chosen array pitch. We mounted the circular nanodisk array on the top of the RIS, reported the field enhancement and the absorption rate, and analyzed the characteristics of the proposed IR absorber.

### 3.1. Circular Nanodisk on SiO_2_ Substrate

**Figure 2:**
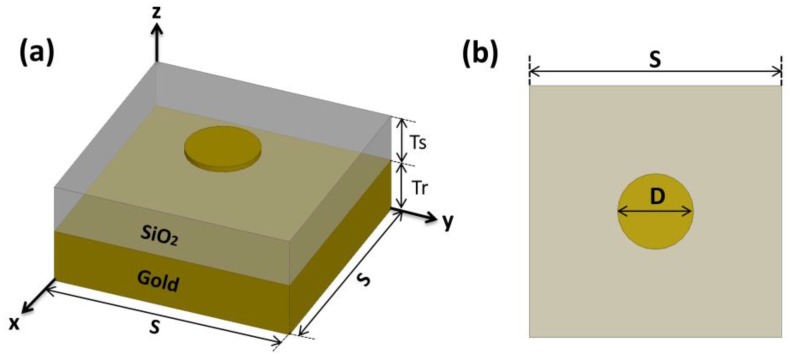
(**a**) Schematic view of the circular nanodisk mounted on the SiO_2_ grounded substrate with T_s_ and T_r_ of 40 and 200 nm, respectively. (**b**) Top view (the x–y plane) with a circular nanodisk diameter (D) of 235 nm and a substrate size (S) of 1.3 µm.

A single circular nanodisk was designed over a SiO_2_ grounded substrate in the radiation boundary. A schematic view of the design is shown in Figure 2. The thicknesses of the gold reflector (T_r_) and disk, and the substrate size (S) were fixed at 200 nm, 10 nm, and 1.3 µm, respectively. An x-polarized plane wave (E_0_ = 1 V/m) was illuminated normally from above the structure, the electric field (E) 1 nm below the edge of the nanodisk was calculated, and E/E_0_ was used for a field enhancement factor [21,41]. To accurately calculate the field enhancement factor, a 10 nm × 10 nm × 10 nm SiO_2_ box enclosing the field calculation point was inserted, and mesh sizes smaller than 1 nm were used. With this simulation setup, we found that 235 nm for the disk diameter (D) and 40 nm for the SiO_2_ thickness (T_s_) provided the maximum field enhancement at 230 THz.

**Figure 3:**
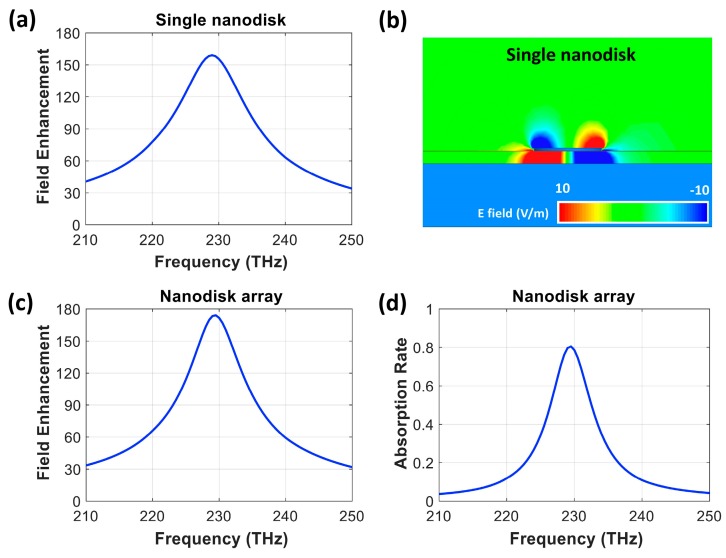
The performance of the single nanodisk and the nanodisk array on the grounded SiO_2_ substrate. (**a**) The electric field enhancement of the single nanodisk with a disk diameter (D) of 235 nm and a SiO_2_ thickness (T_s_) of 40 nm. (**b**) The z component of the electric field distribution (E_z_) in the x–z plane along the disk’s central axis. (**c**) The electric field enhancement and (**d**) the absorption rate of the nanodisk array with an antenna pitch size (P) of 1 µm.

Figure 3a shows the electric field enhancement of the single nanodisk on the grounded SiO_2_ substrate versus frequency. A maximum field enhancement of 159 occurred at 229 THz. We avoided tuning the structure at a nanometer level to realize the resonance at precisely 230 THz. Figure 3b displays the z component of electric field distribution (E_z_) in the x–z plane along the disk’s central axis, indicating that the strong electric fields were confined to the interface between the edges of the nanodisk and the SiO_2_ substrate. This electric field confinement was due to out-of-plane coupling between the nanodisk and the gold reflector [2,3,21].

Using the single nanodisk with maximum field enhancement at 229 THz, we designed its two-dimensional array with master and slave boundaries along the x−z and y–z planes of the simulation boundary. An antenna pitch size (P) of 1 µm produced peak field enhancement at 230 THz, and this result can be attributed to in-phase coupling between the diffraction mode from the array and the LSPPs from the individual nanodisk. This pitch size with the given disk diameter is equivalent to a filling factor of 4.33%, which follows the trend from previous studies [22,28,29,30]. The electric field enhancement and absorption rate of the nanodisk array on the grounded 40 nm thick SiO_2_ substrate with an optimum pitch are presented in Figure 3c,d. The nanodisk in the array structure exhibited resonance near 230 THz with a higher field enhancement factor of 174, compared with a value of 159 from the single nanodisk. The absorption rate of 81% at 230 THz is not perfect because the disk was designed to achieve high field enhancement, and the magnetic resonance was not strong enough to realize impedance matching with air.

### 3.2. Reactive Impedance Surface Design

**Figure 4:**
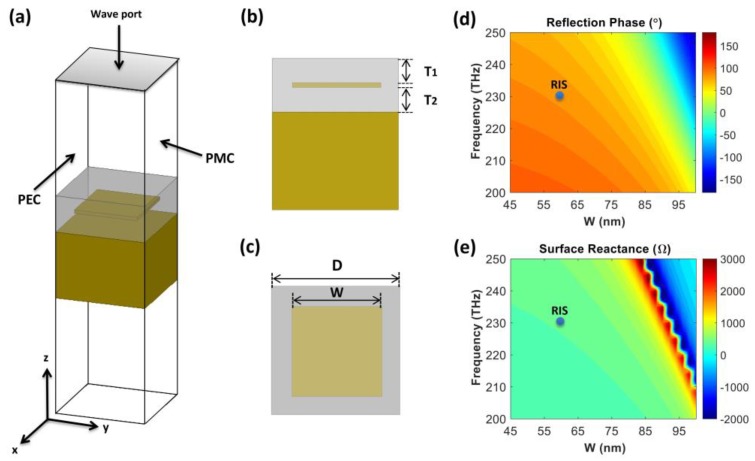
(**a**) Schematic view of the metallic patch unit cell with the perfect electric conductor (PEC) and perfect magnetic conductor (PMC) boundary conditions and wave port excitation. The integration line (x-direction) in the wave port is perpendicular to the PEC boundary and parallel to the PMC boundary. (**b**) Side view (y–z plane) with dimensions of T_1_ = T_2_ = 50 nm. (**c**) Top view (x–y plane) with the patch periodicity (D) and patch width (W). (**d**) Reflection phase and (**e**) surface reactance of the RIS as a function of patch width (W) and frequency (D = 125 nm).

We designed a RIS that will be integrated with a nanodisk array to achieve perfect absorption and high electric field enhancement. The RIS consisted of a square patch array on a 50 nm thick SiO_2_ grounded substrate (T_2_). A 50 nm thick layer of SiO_2_ was placed as a spacer (T_1_) between the patch array and the top of the structure. The top surface of the SiO_2_ spacer was used as a reference plane to calculate the phase of the reflection coefficient, and the nanodisk array was then mounted on that. A schematic view and the detailed simulation setup are presented in Figure 4a–c. The RIS was designed to provide a 90° reflection phase at a resonant frequency of 230 THz because the surface reactance is dominant over surface resistance. First, we set the patch periodicity (D) at 125 nm to fit under the optimum pitch (1 µm) of the nanodisk array and tuned the patch width (W) to realize a 90° reflection phase. In the unit cell simulation for the RIS, an infinite array of metallic patches was modeled using the perfect electric conductor (PEC) and perfect magnetic conductor (PMC) boundary in the y–z and x–z planes of the simulation boundary, and a wave port was used to determine the magnitude and phase of the reflection coefficient of the patch. The surface reactance ($Z_{surf})$ of the RIS was then determined from the complex reflection coefficient (Γ) using the equation $Z_{surf}=\left[\left(1+\Gamma \right)/\left(1-\Gamma \right)\right]\times Z_{0}$, where $Z_{0}$ is the characteristic impedance of the vacuum.

Figure 4d,e depict the phase of reflection coefficient and surface reactance as a function of patch width (W) and frequency, with a fixed patch periodicity (D) of 125 nm. The phase of the reflection coefficient and surface reactance changed from −100° to +100° and from −2000 to 3000 Ω, respectively. We marked points that provided a 90° reflection phase at 230 THz with blue dots and found that a width of 60 nm meets the condition, and the corresponding surface reactance was 405 Ω.

### 3.3. Circular Nanodisk on Reactive Impedance Surfaces

**Figure 5:**
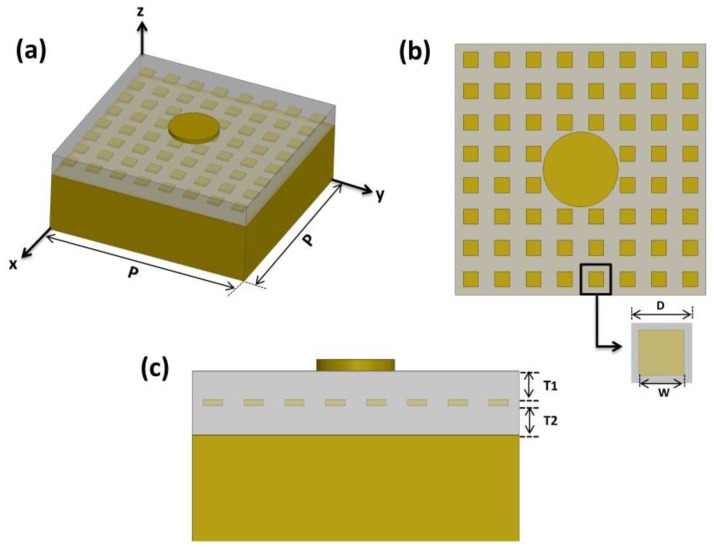
(**a**) Schematic view of the nanodisk mounted atop 8 × 8 reactive impedance surface (RIS) patches. (**b**) Top view (the x–y plane) of the structure with the patch width (W) of 60 nm and the patch periodicity (D) of 125 nm for the RIS patch array. (**c**) Side view (the y–z plane) with T_1_ = T_2_ = 50 nm for thicknesses of the SiO_2_ spacers on top and bottom of the patch array.

Finally, the nanodisk array was integrated with a metallic patch array that functioned as the RIS. The nanodisk was mounted atop the patch array with a 50 nm thick SiO_2_ spacer (T_1_). The SiO_2_ thickness between the patch array and gold reflector (T_2_) was set to 50 nm. Figure 5 shows a schematic view of the circular nanodisk unit cell combined with the RIS. The overall area of the combined unit cell was 1 µm × 1 µm, and the nanodisk was mounted on 8 × 8 RIS patches with a 60 nm patch width (W) and 125 nm periodicity (D).

**Figure 6:**
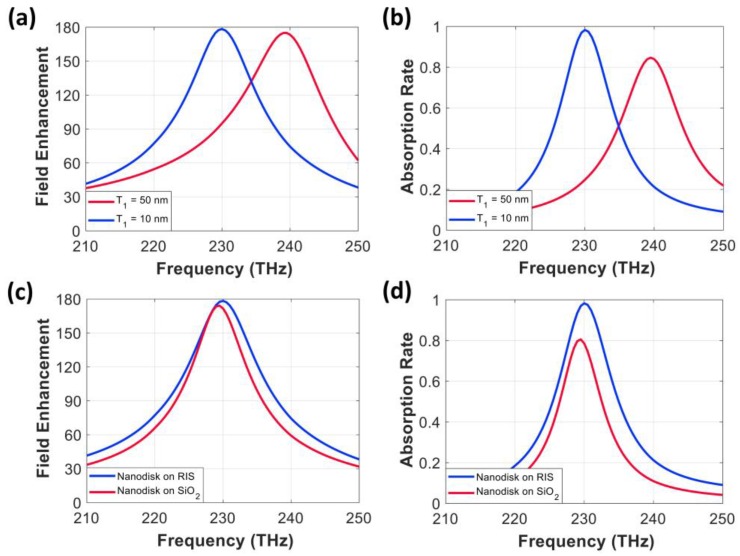
(**a**) Electric field enhancement and (**b**) absorption rate of the nanodisk arrays combined with the 60 nm wide RIS patch array with T_1_ = 50 nm and T_1_ = 10 nm for the SiO_2_ thickness on top of the RIS patch. Note that T_2_ is fixed with 50 nm. The performance comparison between the circular nanodisk on the RIS (T_1_ = 10 nm and T_2_ = 50 nm) and the grounded 40 nm thick SiO_2_ substrate in terms of (**c**) electric field enhancement and (**d**) absorption rate.

The field enhancement and absorption rate of the nanodisk array combined with the 60 nm wide RIS patch and a value of 50 nm for both T_1_ and T_2_ are shown in Figure 6a,b (red color). We found that the addition of the RIS patch array under the nanodisk increased the resonant frequency to 239 THz, while the field enhancement and the absorption rate were 175 and 85%, respectively. The imperfect absorption at 239 THz can be attributed to the mismatch between the surface impedance of the structure and the characteristic impedance of the vacuum. We calculated the surface impedance of the integrated structure and found that surface impedance was 244–j175Ω at 239 THz, which has a lower resistance than the 377 Ω of the vacuum. We also concluded that the surface inductance from the RIS patch (T_1_ = T_2_ = 50 nm and W = 60 nm) did not cancel capacitance from the nanodisk at 230 THz. To lower the resonant frequency to 230 THz, we reduced the spacer thickness (T_1_), which increases the capacitance from the disk and, at the same time, decreases the inductance of the RIS.

Finally, a T_1_ value of 10 nm provided resonance at 230 THz with a high field enhancement value of 180 and almost perfect absorption (98%) at 230 THz (blue line), as shown in Figure 6a,b. From the simulation, the integrated structure with T_1_ of 10 nm produced a surface impedance of 377 + j39 Ω at 230 THz, which shows a similar resistance with the characteristic impedance of the vacuum. We further calculated the surface reactance of the patch array for the 60 nm wide RIS (T_1_ = 10 nm and T_2_ = 50 nm) and verified that the surface reactance value became 225 Ω, which is lower than 405 Ω from the RIS (T_1_ = 50 nm and T_2_ = 50 nm) at 230 THz. Because the reactance values from the RISs were calculated from the wave port, a disk sitting near a RIS would experience a different reactance. To estimate the effective inductance of the RIS that compensated the capacitance of the disk, we calculated the capacitance of the disk using an equation (C_m_ = c_1_ε_d_ε_0_A/d) for a parallel plate capacitor with a modification (adding c_1_), which reflects the LSPP effect [44]. The capacitance (C_m_) of the circular disk (D = 235 nm) sitting on the ground with 70 nm thick SiO_2_ was 4.4 aF. Here, a fitting constant (c_1_) of 0.2 was used to consider nonuniformly distributed charges on the disk. C_m_ indicates capacitance from one edge of the disk, so the overall capacitance of the disk should be 2C_m_ (8.8 aF). On the other hand, the inductance from the disk itself was neglected because the level was near fH. The final 8.8 aF capacitance of the disk requires 54 fH inductance to maintain the resonance at 230 THz. Because 225 Ω at 230 THz from the optimum RIS is equivalent to 156 fH, we can estimate that about one-third of the inductance calculated from the wave port is effective in the RIS-combined structure. The calculated capacitance of the circular disk is also from approximation; thus, the effective inductance from the RIS can be varied. However, it is important to note that a significant portion of inductance from the RIS is needed to effectively cancel the capacitance of the disk and realize almost perfect absorption at 230 THz.

Figure 6c,d compare field enhancement and absorption rate values between the optimum RIS-integrated structure (T_1_ = 10 nm and T_2_ = 50 nm) and the nanodisk array on the grounded 40 nm thick SiO_2_ substrate. The peak values near 230 THz for both cases were 180 versus 174 in the field enhancement and 98% versus 81% in the absorption rate. This result means that only a RIS-integrated absorber can achieve near-perfect absorption along with high field enhancement (>100), even with a thick SiO_2_ spacer. We further calculated a field intensity ratio at the hotspot with and without the nanodisk in the RIS-combined absorber and found that the value was 140. Due to a slightly enhanced field intensity of 1.29 V/m from the RIS in the absence of the disk, the ratio became lower than the field enhancement factor (180), but it was still higher than 100.

Furthermore, Figure 6d shows that the nanodisk array on the RIS had a wider absorption bandwidth compared with the nanodisk array on the grounded SiO_2_ spacer due to an increased overall volume of SiO_2_ that maintains high near-field coupling. The absorption bandwidths of the nanodisk array on the RIS and the grounded SiO_2_ substrate were 4.13% and 3.47% (based on full width at half maximum), respectively. Finally, the final RIS-integrated MIM absorber outperformed the reported ultrathin spacer-based circular nanodisk structures, which exhibited the maximum field enhancement near 85 with an absorption rate of 95% [21,22,23,31,32,33]. The field enhancement of 180 (E/E_0_) from the proposed absorber can be converted to a surface-enhanced Raman scattering (SERS) factor which is used for IR spectroscopy applications. Because the SERS factor is proportional to |E/E_0_|^4^, the proposed absorber will show nearly 20 times higher SERS factor compared to the other absorbers. Furthermore, the RIS-combined absorber can be used for a single molecule detection as its SERS enhancement of 10^9^ is higher than the threshold levels of 10^7^–10^8^ [45,46].

**Figure 7:**
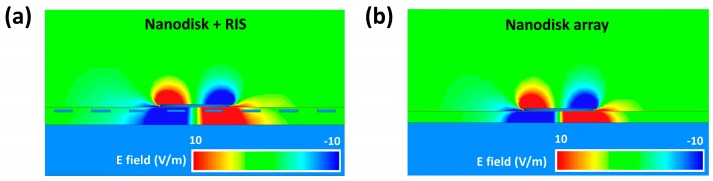
The z component of the electric field (E_z_) in the x−z plane along the disk’s central axis for (**a**) the nanodisk combined with the 60 nm wide RIS patch and SiO_2_ spacers (T_1_ = 10 nm and T_2_ = 50 nm) and (**b**) the nanodisk array on the grounded 40 nm thick SiO_2_ substrate.

To understand the coupling mechanism between the nanodisk array and the RIS patch array, we calculated the z component of the electric field (E_z_) in the x−z plane along the disk’s central axis. Figure 7a shows that the coupling between the LSPPs and the array diffraction was not disturbed due to the RIS patch array, and strong fields were maintained in a wider area between the disk and the reflector compared with the fields from the nanodisk array in Figure 7b. From this analysis, we concluded that the higher field enhancement and absorption rate from the nanodisk array combined with the RIS patch could be attributed to the strongly coupled field in a larger volume.

### 3.4. Polarization Independence of IR Absorber Based on RIS

We further investigated the polarization sensitivity of the proposed IR absorber to demonstrate its polarization independence. Due to the circular shape of the nanodisk and the square shape of the metal patch array for the RIS, we expected the same optical response for x- and y-polarized incident waves. In numerical simulations, the circular disk array integrated with the RIS patch (W = 60 nm, T_1_ = 10 nm, T_2_ = 50 nm) was additionally illuminated with a y-polarized incident plane wave in a normal direction from the top.

**Figure 8:**
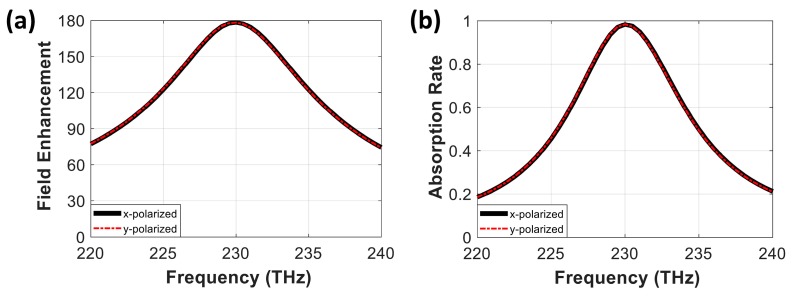
Demonstration of polarization independence of the proposed absorber. (**a**) Electric field enhancement and (**b**) absorption rate of the proposed absorber with different types of polarization. The solid black line and red dashed line represent x- and y-polarized incident waves, respectively.

Figure 8 shows the electric field enhancement and the absorption rate of the integrated absorber with x- and y-polarized incident plane waves in a normal direction. The exact overlaps of the parameters indicate the polarization independence of the proposed absorber, although the RIS patch was combined. The symmetrical arrangement of the RIS patch array beneath the circular nanodisk allowed for stable near-field coupling for both x- and y-polarized incident field cases. This polarization-independent feature from the RIS-integrated nanodisk absorber would therefore guarantee robust performance, even with unpolarized or any linearly polarized incident waves.

### 3.5. Discussion on Fabrication and Experiment of RIS-combined IR Absorber

Similar to other nanostructures, nanometer-scaled gold shapes (<100 nm) for the disk and the RIS can be patterned using e-beam lithography (EBL) or focused ion beam (FIB) milling, and the SiO_2_ layers between metallic structures can be deposited using atomic layer deposition (ALD) [47,48,49,50,51]. In the multilayered RIS-combined nanodisk array, depositing gold inside SiO_2_ layers and alignment between the disk and the RIS patch would be challenging tasks. We found a solution where gold patterning inside a SiO_2_ layer, such as a buried nanoantenna, was realized by filling gold in an engraved SiO_2_ substrate etched by reactive ion etching (RIE) [49]. Also, the alignment between metallic patterns in two layers was resolved with the EBL technique using alignment marks [52].

In experiments, the absorption rate can be measured using Fourier transform infrared (FTIR) spectrometer along with a microscope [21]. Furthermore, the field enhancement can be measured using near-field scanning optical microscopy (SNOM) with scattering-type scanning methods [53].

## 4. Conclusions

In this study, we proposed a polarization-independent IR absorber based on a circular nanodisk array combined with a RIS to achieve high electric field enhancement and perfect absorption. The proposed absorber, with a filling factor of 4.33%, produced an electric field enhancement value of 180 and a near-perfect absorption rate of 98% at 230 THz. These values were higher than the 174 and 81% associated with a circular nanodisk array on a grounded 40 nm thick SiO_2_ substrate with the same filling factor. The proposed MIM absorber also outperformed other reported ultrathin spacer-based MIM absorbers, for which the field enhancement saturates near 85. Furthermore, we demonstrated a polarization-independent property of the proposed absorber by illuminating x- and y-polarized incident waves. Due to the symmetrical arrangement of the circular disk and patches for the RIS, the proposed absorber was stable with the polarization change of the incident wave, confirming the merits of the proposed absorber. In applications, a RIS-integrated MIM absorber with a superior field enhancement can be used to enhance the sensitivity of sensors, such as LSPR sensors and surface-enhanced IR spectroscopy.
